# Simulation-Based Design and Machine Learning Optimization of a Novel Liquid Cooling System for Radio Frequency Coils in Magnetic Hyperthermia

**DOI:** 10.3390/bioengineering12050490

**Published:** 2025-05-04

**Authors:** Serhat Ilgaz Yöner, Alpay Özcan

**Affiliations:** 1 Institute of Biomedical Engineering, Boğaziçi University, İstanbul 34342, Türkiye; 2 Department of Biomedical Equipment Technology, Junior College, Acıbadem Mehmet Ali Aydınlar University, İstanbul 34752, Türkiye; 3 Magnetic Medical Devices Laboratory, Boğaziçi University, İstanbul 34342, Türkiye; 4 Electrical and Electronics Engineering Department, Boğaziçi University, İstanbul 34342, Türkiye; 5 Systems Science and Mathematics Laboratory, Boğaziçi University, İstanbul 34342, Türkiye; 6 Center for Targeted Therapy Technologies (CT3), Boğaziçi University, İstanbul 34342, Türkiye

**Keywords:** magnetic hyperthermia, radio frequency, electromagnetic heating, skin effect, liquid cooling, microchannel, finite element method, machine learning optimization, Gaussian process regression

## Abstract

Magnetic hyperthermia is a promising cancer treatment technique that relies on Néel and Brownian relaxation mechanisms to heat superparamagnetic nanoparticles injected into tumor sites. Under low-frequency magnetic fields, nanoparticles generate localized heat, inducing controlled thermal damage to cancer cells. However, radio frequency coils used to generate alternating magnetic fields may suffer from excessive heating, leading to efficiency losses and unintended thermal effects on surrounding healthy tissues. This study proposes novel liquid cooling systems, leveraging the skin effect phenomenon, to improve thermal management and reduce coil size. Finite element method-based simulation studies evaluated coil electrical current and temperature distributions under varying applied frequencies, water flow rates, and cooling microchannel dimensions. A dataset of 300 simulation cases was generated to train a Gaussian Process Regression-based machine learning model. The performance index was also developed and modeled using Gaussian Process Regression, enabling rapid performance prediction without requiring extensive numerical studies. Sensitivity analysis and the ReliefF algorithm were applied for a thorough analysis. Simulation results indicate that the proposed novel liquid cooling system demonstrates higher performance compared to conventional systems that utilize direct liquid cooling, offering a computationally efficient method for pre-manufacturing design optimization of radio frequency coil cooling systems in magnetic hyperthermia applications.

## 1. Introduction

Magnetic hyperthermia (MH) is a promising cancer treatment technique that utilizes the unique properties of magnetic nanoparticles (MNPs) to achieve targeted heating of tumor sites [1,2,3]. The fundamental physics of MH involves the injection of these MNPs directly into the tumor, followed by the application of an alternating magnetic field. Within the approximate 0.1–3 MHz radio frequency (RF) range, alternating magnetic fields trigger heat generation in MNPs through Néel relaxation (magnetic moment reorientation within the particle) and Brownian relaxation (physical particle rotation) mechanisms in the surrounding fluid [4,5]. This localized heat generation leads to controlled thermal damage of cancer cells. The clinical significance of MH lies in its potential to offer a minimally invasive and selective treatment option for various cancers, including challenging malignancies like glioblastoma, wherein conventional treatments face limitations [6,7].

A key challenge in advancing MH therapy is the precise control of heat generation [8,9]. Addressing this challenge necessitates ensuring that therapeutic heating is primarily focused on tumor sites through MNP—RF interactions, while minimizing unintended temperature increases in surrounding healthy tissues that may arise from excessive heating of the RF coil [10] or from the accumulation of non-targeted or rapidly dispersing MNPs post-injection, which can induce off-target thermal damage [11]. Resolving the RF coil-related challenge should not only enhance the efficacy of MH but also contribute to advancements in broader thermal therapy applications and impact other medical modalities employing RF energy, leading to improvements across various therapeutic techniques. Several studies have focused on the targeted delivery of MNPs to enhance therapeutic selectivity. In one study, MNPs were developed to selectively target and eradicate ectopic endometriotic tissue in mice, demonstrating the potential to achieve high heating efficiency and specificity [12]. In another study, trimagnetic MNPs were designed for prostate cancer therapy, demonstrating enhanced intracellular MH with minimal invasiveness and toxicity [13]. Additionally, one study explored the use of MNPs to increase the efficacy of antibiotics for lung infections in cystic fibrosis patients, emphasizing the advantages of targeted drug delivery and reduced systemic toxicity [14]. Collectively, these studies underscore the critical importance of ongoing efforts to enhance the selectivity of MH therapy, ensuring precise targeting and minimizing off-target effects to maximize therapeutic benefits while minimizing harm to healthy tissues. However, compared to the extensive research on MNP optimization, studies focusing on RF coil cooling system design and optimization for enhanced therapeutic selectivity remain relatively limited, making the development of novel approaches in this domain particularly impactful.

The integration of machine learning (ML) techniques presents a potent strategy for optimizing MH systems. ML models are capable of analyzing complex datasets, encompassing MNP characteristics, coil geometry, material properties, operating parameters, and simulated or experimental electromagnetic and thermal distributions, to accurately and rapidly predict system behavior and identify optimal design configurations [15]. This predictive capability facilitates pre-manufacturing optimization of both MNP synthesis and RF coil design, substantially reducing the costly and time-consuming process of physical prototyping [16]. Furthermore, ML algorithms can provide precise forecasting of thermal distribution deviations under varying experimental conditions, enabling more robust control of heat generation and ultimately leading to more reliable and effective MH therapies.

This study proposes a novel liquid cooling system for RF coils in MH and compares its performance with two conventional cooling systems, integrating finite element method (FEM) analysis with ML-based predictions, enabling pre-manufacturing design optimization and precise forecasting of performance deviations under experimental conditions. To systematically evaluate the proposed design, a performance index was developed and modeled using both Support Vector Regression (SVR)- and Gaussian Process Regression (GPR)-based ML models, incorporating applied frequency, coil wire temperature, and cooling water flow rate parameters. The following sections detail the materials, methods, and validation framework used to assess the system’s effectiveness.

## 2. Materials and Methods

**Figure 1:**
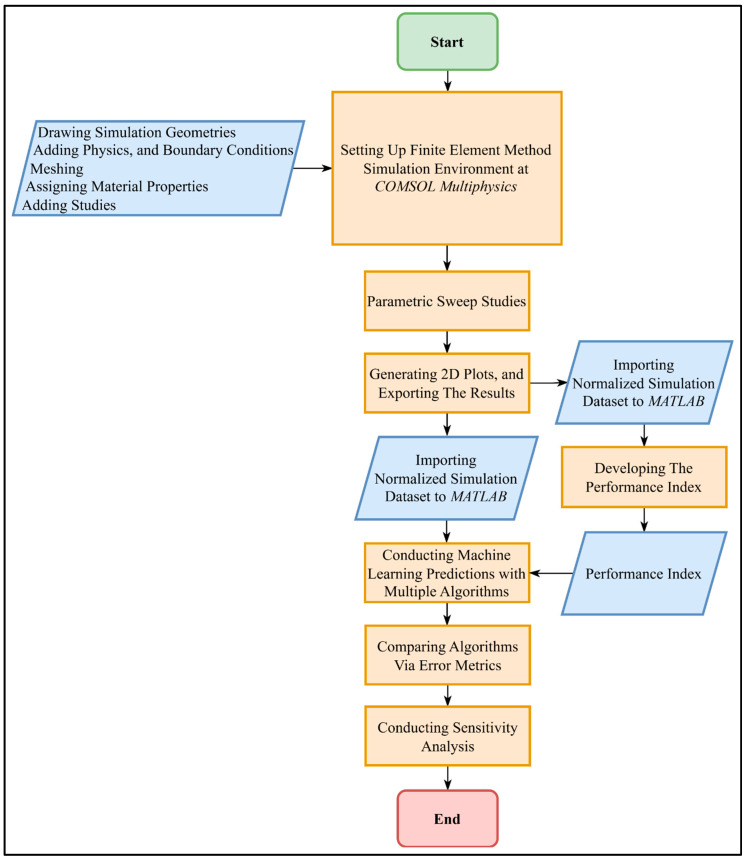
Overview of the methodological workflow in this study, presented in the form of a flowchart. (Rectangle and parallelogram shapes represent system processes and inputs, respectively).

This study follows a structured workflow beginning with FEM simulations and progressing through performance index formulation, ML modeling, model evaluation, and sensitivity analysis. The overall methodological approach is summarized in Figure 1 in the form of a flowchart.

### 2.1. Simulations

Three different coil cooling systems were simulated using the FEM in COMSOL Multiphysics Software 5.5 [17,18,19]: a conventional passive cooling system that transfers heat through stagnant air via natural convection, a conventional liquid cooling system wherein the coolant flows axially along the coil axis and contacts the outer surface of the wires, and a novel liquid cooling system wherein the coolant flows through a microchannel inside the wires, axially along the wire axis and contacts the inner wire surface. The conventional passive cooling system served as the electromagnetic benchmark, assessing the coil wires’ current distribution influenced by the frequency of electromagnetic fields, while the conventional liquid cooling system served as the thermal benchmark, evaluating the system’s temperature, which is affected by heat dissipation from the coil wires to the cooling liquid.

The 2D axisymmetric option was chosen as the simulation space due to its computational efficiency [20] and ability to accurately capture the axisymmetric geometry of the coil cooling system. The Electrical Circuits module was used to supply 25 A peak alternating current (AC) to the coil wires, generating magnetic fields in space via the Magnetic Fields module, relying on Poisson’s equation,

(1) E=−𝛻ϕ

where E is the electric field, 𝛻 is the Del operator, and ϕ is the electric potential. The expression 𝛻ϕ gives the rate and direction of change in the electric field at each point in space, and the negative sign (−) indicates that the electric field points in the direction of the greatest decrease in electric potential. Accordingly, the Magnetic Fields module uses Ohm’s Law within the context of electromagnetics to compute the current density norm,

(2) J=σE

where σ is the electrical conductivity of the material. The Magnetic Fields module was one-way coupled with the Heat Transfer in Solids module to compute the electromagnetic heating,

(3) Q=J·E

**Figure 2:**
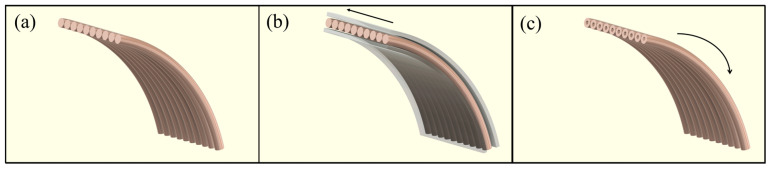
Different coil cooling systems: (**a**) the conventional passive, (**b**) the conventional liquid with DLC, and (**c**) novel liquid with various microchannel radii of 0.25, 0.30, and 0.35 mm. (Arrows indicate cooling liquid flow directions; (**b**) semi-transparent regions around coil wires indicate the outer shell of the DLC system.)

COMSOL computes the final temperature, T, for each point in the simulation space, considering the steady-state scenario, with the following expression:

(4) 0=𝛻·(k𝛻T)+Q

where k is the thermal conductivity of the material, 𝛻T is the gradient of temperature, which gives the rate of change in temperature at a point in space, indicating the direction of the steepest temperature increase. The expression 𝛻·(k𝛻T) is the divergence of the heat flux, describing how heat is diffusing through the material. The combined use of these modules ensured a detailed replication of the magnetic and thermal physics. The same coil geometry, with a 22 mm radius, 12.4 mm length, and 10 wire turns was used with 1.2 mm diameter wires to test the performance of both cooling systems inside a cylindrical space with a 500 mm radius and 1000 mm length. Copper’s material properties and coil boundary conditions were assigned to the coil wires to fully replicate electrical, magnetic, and thermal characteristics. To prevent the accumulation of thermal energy within the confined cylindrical space and maintain simulation realism, a convective heat flux boundary condition was applied to the domain’s edges using the external natural convection option at 1 atm absolute pressure. Additionally, the cylindrical space was defined as an ideal gas with the material properties of air. The conventional liquid cooling system utilizes direct liquid cooling (DLC) [21,22], where the coolant flows axially along the coil axis, surrounding the coil wires with a thickness of 1.5 mm. In contrast, the novel liquid cooling system directs the coolant through an internal microchannel running parallel inside the wires. Three versions of this system were designed, each featuring a different microchannel radius: 0.25, 0.30, and 0.35 mm. Distilled water was selected as the cooling fluid, and material properties were assigned to the relevant domains of both the conventional and novel liquid cooling systems. All material properties are provided in Table S1 of the Supplementary Materials with values sourced from reference [23], and Figure 2 illustrates all coil cooling systems.

All simulation geometries were meshed using free triangular elements, with minimum and maximum mesh sizes of 0.02 and 10 mm, respectively. Additionally, a boundary layer was assigned to the edges of the coil wires, with the number of boundary layers set to 12 and the boundary layer stretching factor set to 1.2. This approach enhanced the mesh quality, enabling high-resolution current distribution maps to be obtained. The initial step of the simulations involved Frequency Domain Analysis to compute magnetic fields and current distributions, followed by Time-Dependent Analysis to compute temperatures. Simulations were conducted at an ambient starting temperature of 20 °C and solved at the 60-minute mark for different combinations of applied frequency, $f_{a}$, and water flow rate, $v_{wf}$, parameters. The applied frequency ranged from 0.1 to 2.9 MHz, with a step size of 0.2 MHz, while the water flow rate varied from 0.1 to 0.5 m/s, with a step size of 0.1 m/s, resulting in 75 separate simulations for each coil cooling system. A domain probe was defined in COMSOL Multiphysics to evaluate current distributions for electromagnetic benchmarking by computing the spatial maximum current density normal,

(5) $$smJ=\underset{V}{\max}\left(J\right)A/m^{2}$$

where J is the current density vector component perpendicular to the wire surface, and V is the spatial volume over which the maximum current density is computed. The 2D slice plots of the spatial maximum current density normal were generated for all designs at 0.1, 1.5, and 2.9 MHz applied frequencies. Another domain probe was defined to compute the spatial maximum temperature for thermal benchmarking,

(6) $$smT=\underset{V}{\max}\left(T\right){}^{\circ}C$$

where T is the temperature of the coil wires at any given point. The 2D slice plots of the spatial maximum temperature were generated for all designs with liquid cooling systems at 0.1 and 2.9 MHz applied frequencies, combined with 0.1 and 0.5 m/s water flow rates. Coil wattage as a function of applied frequency was computed in simulation, and a related line plot was generated for all coil designs, to showcase thermal power output trends across the operational frequency range. The resulting dataset consisting of the spatial maximum current density normal and spatial maximum temperature was then imported into MATLAB R2022A [24] for further analysis.

### 2.2. Performance Index

All variables, applied frequency, water flow rate, spatial maximum current density normal, and spatial maximum temperature, were normalized between 0 and 1, corresponding to $nf_{a}$, $nv_{wf}$ nsmJ, and nsmT, respectively, ensuring comparability across different scales, with 0 representing the lowest observed value and 1 the highest.

(7a) $$nf_{a}=\frac{f_{a}-f_{a,min}}{f_{a,\;\;max}-f_{a,min}}$$

(7b) $$nv_{wf}=\frac{v_{wf}-v_{wf,min}}{v_{wf,\;\;max}-v_{wf,min}}$$

(7c) $$nsmJ=\frac{smJ-smJ_{min}}{smJ_{max}-smJ_{min}}$$

(7d) $$nsmT=\frac{smT-smT_{min}}{smT_{max}-smT_{min}}$$

These normalizations allow for an unbiased performance index calculation,

(8) $$PI=\frac{1+nf_{a}}{1+nsmT+nv_{wf}}$$

where the numerator accounts for the applied frequency, which enhances therapeutic effectiveness by increasing the energy transfer [25,26]. The denominator accounts for the spatial maximum temperature and the water flow rate, as higher coil wire temperatures reduce the selectivity of MH thermal therapy [27,28], and increased water flow rates impose higher power demands on the pumping system. Using summation in the denominator ensures that no single factor dominates, maintaining a balanced assessment. The addition of 1 to the denominator prevents division by 0, ensuring numerical stability, while the 1 in the numerator ensures that the performance index remains sensitive to changes in either the normalized spatial maximum temperature or the normalized water flow rate, even at the lowest normalized applied frequency (0) [29,30]. This formulation provides a normalized, stable, and intuitive metric for comparing different cooling system designs.

### 2.3. Machine Learning Predictions

The dataset was modeled using both SVR and GPR in MATLAB to evaluate multiple relationships within the system. Specifically, the relationship between the applied frequency and the normalized spatial maximum current density normal was modeled separately. Additionally, the relationship between the applied frequency, water flow rate, and normalized spatial maximum temperature was analyzed. Lastly, both models were used to predict the performance index as a function of the applied frequency and water flow rate. Hold-out validation [31] was conducted using an 80–20% split for training and testing, respectively. Model performance was assessed using adjusted R^2^ as the error metric to ensure a fair comparison of predictive accuracy [32].

Following model training, a sensitivity analysis [33] was performed to evaluate the relative influence of the applied frequency and water flow rate in the GPR and SVR models of the predicted performance index outcomes, providing insights into parameter significance within the system. Sensitivity analysis was conducted using a One-at-a-Time (OAT) method, where the applied frequency and water flow rate were systematically varied by ±10% to assess their influence on predicted outcomes. Additionally, the ReliefF algorithm [34,35] was applied to rank the relative importance of the applied frequency and water flow rate in the GPR and SVR models of the performance index.

## 3. Results

**Figure 3:**
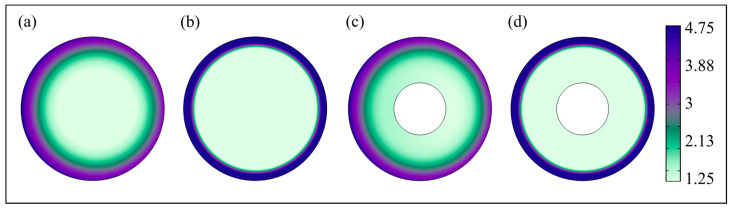
Basic simulation results comparing 2D slice plots of the spatial maximum current density normal, smJ, in solid coil wires and coil wires with a microchannel radius of 0.25 mm at 0.1 and 2.9 MHz applied frequencies. Solid coil wire results are shown at (**a**) 0.1 and (**b**) 2.9 MHz, while results for coil wires with a microchannel radius of 0.25 mm are shown at (**c**) 0.1 and (**d**) 2.9 MHz. (The color bar on the right indicates the magnitude of current density normal ×10^7^ A/m^2^.)

Electromagnetic benchmarking simulation results in Figure 3 indicate that the current density normal distributions in solid coil wires and coil wires with a microchannel radius of 0.25 mm are highly similar across the applied frequency range. Both wire configurations exhibit comparable current density magnitudes and spatial distributions, with no significant deviations observed between them, at the same applied frequency.

The key thermal benchmarking simulation results in Table 1 show that, compared to the novel liquid cooling systems, the spatial maximum temperature in the conventional liquid cooling system is slightly higher at 0.1 MHz applied frequency with water flow rates of 0.1 and 0.5 m/s (bolded entries 24.964 and 23.172 °C) and noticeably higher at 2.9 MHz applied frequency with the same water flow rates (bolded entries 52.050 and 40.816 °C), indicating poorer thermal performance. Figures and tables relating to the electromagnetic benchmarking and thermal benchmarking intermediate steps are provided under the relevant sections in the Supplementary Materials.

The performance index gradually decreases as the applied frequency decreases and the water flow rate increases, reaching minimum values of 0.480, 0.499, 0.500, and 0.500, as bolded entries in Table 2, Table 3, Table 4 and Table 5, respectively, for each cooling system. Conversely, the performance index steadily increases as the applied frequency increases and the water flow rate decreases, reaching maximum values of 1.000, 1.505, 1.566, and 1.614, as bolded entries in Table 2, Table 3, Table 4 and Table 5, respectively. The only exceptions occur in specific cases of the conventional liquid cooling system, where a decrease in the performance index is observed for the applied frequency–water flow rate combinations of 0.1 to 0.5 MHz at 0.1 m/s and 0.1 to 0.3 MHz at 0.2 m/s, as bolded entries at Table 2. Overall, the conventional liquid cooling system has the lowest performance index (bolded 0.480), while the novel liquid cooling system with a microchannel radius of 0.35 mm has the highest performance index (bolded 1.614). For further details on the developed performance index, see the relevant section in the Supplementary Materials.

The SVR predictions yielded poor fits across all cases, with the adjusted R^2^ ranging from 0.9177 to 0.9777, whereas GPR predictions performed significantly better, ranging from 0.9990 to 0.9999 with uncertainty estimates at a 95% confidence level. The same error metrics are provided for the performance index predictions in Table 6. See the Electromagnetic Benchmarking, Thermal Benchmarking, and Performance Index Sections in the Supplementary Materials for SVR and GPR model plots and error metrics.

The sensitivity analysis suggests that, for a 10% increase in both parameters, the water flow rate has a slightly higher impact on performance than the applied frequency across all cooling systems. Furthermore, the conventional liquid cooling system is the least affected, while the novel liquid cooling system with a 0.35 mm microchannel radius exhibits the greatest sensitivity to changes in both parameters. The ReliefF algorithm revealed that the applied frequency parameter has a higher weight on the overall performance of the conventional liquid cooling system compared to the water flow rate parameter. In contrast, for the novel liquid cooling systems with microchannel radii of 0.25, 0.30, and 0.35 mm, the water flow rate parameter was found to have a greater impact on performance than the applied frequency parameter. The sensitivity analysis results and the ReliefF feature weights for the SVR and GPR models of the performance index are provided in the Performance Index Section in the Supplementary Materials, and in Table 7, respectively.

## 4. Discussion

Many high-performance coil designs incorporate liquid cooling systems with coolant flowing axially along the coil axis [21,22]. However, the enclosure surrounding such liquid cooling systems occupies valuable space, particularly on the inner side of the coil, reducing the total volume of the gap found in coil designs. These gaps are critical functional volumes in MH coils, where the anatomical region containing the tumor site is positioned during MH therapy. The coil radius can be increased to compensate for the reduction in functional volume caused by a decrease in the radial dimension, resulting in increased coil impedance. This, in turn, leads to a higher power requirement to maintain the initial magnetic field strength [36,37]. Also, literature reports indicate that magnetic nanoparticles like Fe_3_O_4_ and MnFe_2_O_4_, at 2 mg/mL concentrations under clinical field conditions, generate only ~0.75–2 W of thermal energy [38,39]. In contrast, the coil wattage in our system exceeds 150 W at higher frequencies (See Supplementary Materials, Figure S6). This disparity underscores the risk of non-selective heating from coil resistive losses, which can inadvertently warm healthy tissues and compromise therapeutic precision. Thus, implementing effective coil cooling is essential to ensure selective and safe magnetic hyperthermia treatment.

In this study, a novel liquid cooling system utilizing microchannel structures [40] was designed within an FEM simulation environment to operate in a frequency range of 0.1–2.9 MHz and a water flow rate range of 0.1–0.5 m/s. The frequency range was selected based on its prevalence in MH systems [41], ensuring relevance to current biomedical applications. In contrast, the water flow rate range was determined heuristically, taking into account the microchannel [42] dimensions of the cooling system (0.25, 0.30, and 0.35 mm radii) and the need to maintain stable, laminar flow for effective heat dissipation. Although not derived from empirical tuning or experimental optimization, this range was considered a practical starting point for assessing the cooling system’s thermal performance [43,44]. It provides a realistic baseline for future refinement. Distilled water was chosen as a widely used cooling fluid due to its purity, which reduces the risk of scale deposition and maintains consistent thermal performance. This minimizes the impact on heat transfer efficiency, ensuring that the coil’s electromagnetic properties are not compromised by impurities or scaling [45,46]. Results indicate that the proposed novel liquid cooling system (see Figure 2c), in which the coolant flows through a microchannel inside the wires axially along the wire axis and contacts the inner wire surface, can effectively address the design challenge mentioned in the preceding paragraph. This novel design leverages the skin effect phenomenon (the tendency of AC to flow on the surface of the wire, with the current density decreasing with depth as the applied frequency increases [47]) by incorporating a cooling microchannel inside the wires, occupying the electron-free region corresponding to AC resistance (see Figure 3c,d). This approach enhances thermal management compared to the conventional liquid cooling system (see Table 1), while either preserving or reducing the original coil radius. It is important to note that the novel liquid cooling systems with microchannel radii of 0.25, 0.30, and 0.35 mm dissipate heat not only through the microchannel and into the coolant but also via the outer surface of the wires into the surrounding stagnant air. This dual heat dissipation mechanism helps clarify how the novel cooling system outperforms the conventional liquid cooling system. The correct selection of an applied frequency–water flow rate combination is crucial, as an increase in the applied frequency enhances therapeutic effectiveness by increasing the energy transfer [25,26], and an increase in the water flow rate imposes higher power demands on the pumping system. Disruptive effects of the coil wire temperature on the selectivity of MH thermal therapy [27,28] may be used as the deciding factor when selecting the optimum operation point. Traditional systems address the reduced selectivity of MH therapy caused by an increased coil wire temperature by increasing the coolant flow rate or enlarging the coil wire. However, these approaches often overlook the increased power demands on the pumping system [48] and the reduction in functional volume [49], respectively. Therefore, optimizing the applied frequency and water flow rate input parameters is critical for reducing power demands in any form.

The trend of the performance index across all cooling systems (see Table 2, Table 3, Table 4 and Table 5) highlights the critical interplay between the applied frequency and water flow rate. Systems that maintain higher performance indices under increased applied frequencies and reduced water flow rates demonstrate enhanced efficiency, under such less favorable thermal loads. Notably, the novel liquid cooling system with a 0.35 mm microchannel radius consistently outperformed others, especially the conventional liquid cooling system, with a prominent performance index difference of 0.614 under 2.9 MHz applied frequency and 0.1 m/s water flow rate conditions, indicating that the novel cooling microchannel contributes significantly to thermal regulation effectiveness, with additional passive heat dissipation naturally occurring through the outer surface of the wires to the surrounding stagnant air, further improving the overall cooling efficiency of the system. Additionally, the performance gap between the conventional liquid cooling system and the novel liquid cooling system widens with the increasing applied frequency, in favor of the latter. A further increase in the applied frequency is likely to amplify this gap even more, which is promising in terms of the adaptability of the novel liquid cooling system in other RF applications operating at higher frequencies, broadening the significance of this study for future advancements in thermal management across a range of RF technologies. The markedly inferior performance of the conventional liquid cooling system, along with its irregular behavior under certain parameter combinations (performance indices corresponding to the applied frequency–water flow rate combinations of 0.1 to 0.5 MHz at 0.1 m/s and 0.1 to 0.3 MHz at 0.2 m/s, as bolded entries at Table 2), underscores the limitations of traditional cooling approaches under dynamic operating conditions. These findings reinforce the value of the developed performance index as a comparative metric and suggest that fine-tuning structural parameters like the microchannel radius can be a key enabler of thermal performance gains.

**Figure 4:**
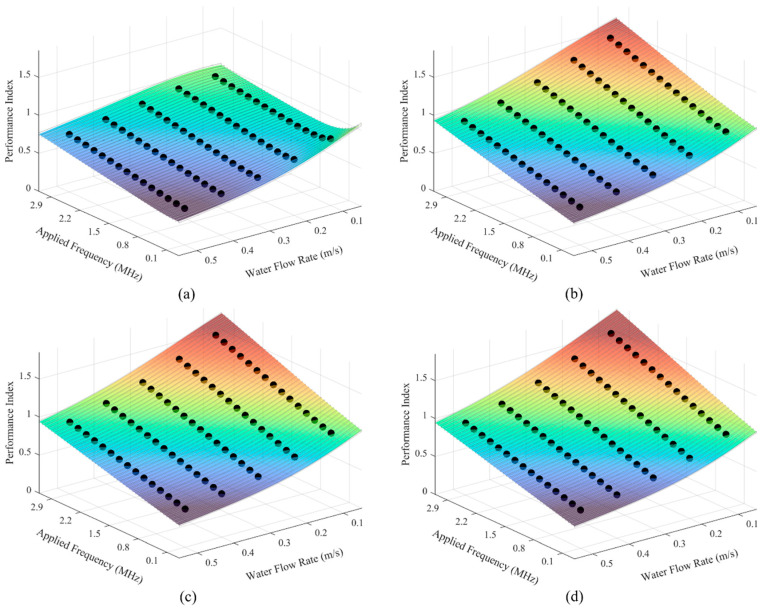
Data and GPR model predictions of applied frequency, $f_{a}$, and water flow rate, $v_{wf}$, vs. performance index, PI, for all liquid cooling systems: (**a**) conventional liquid and (**b**–**d**) novel liquid with microchannel radii of 0.25, 0.30, and 0.35 mm, respectively. (Scatter: data; surface: predicted; the lines encircling the color surface indicate uncertainty estimates at a 95% confidence level).

Two ML models, SVR and GPR, were employed for predictions. SVR was chosen for its flexibility, particularly in handling complex relationships, and its robustness to outliers [50]. On the other hand, GPR was selected for its ability to not only predict values but also quantify the uncertainty associated with those predictions, offering deeper insights into the reliability of the performance index [51]. The use of both models enabled a thorough analysis and comparison of different approaches, allowing for an informed decision on the most appropriate algorithm based on the available data. The results demonstrated that the GPR model provided the most accurate performance index predictions (see Figure 4 and Table 6), and consequently, it was adopted for all subsequent model predictions. In practical applications, deviations in manufacturing precision, operational conditions, and environmental factors can introduce variations in coil liquid cooling system performance compared to simulations. Factors such as material inconsistencies, coolant property fluctuations, flow rate variations, and real-world electrical effects may lead to discrepancies in current density distribution and thermal management efficiency. GPR’s uncertainty estimates provide a means to interpret and anticipate these variations, highlighting regions where deviations are more likely and guiding experimental validation or design refinements [51]. Sensitivity analysis and the ReliefF algorithm were applied to the SVR and GPR predictions of the performance index for a deeper evaluation of system behavior (see Table 7). The sensitivity analysis results indicate that the water flow rate has a slightly greater impact on performance than the applied frequency across all cooling systems. Meanwhile, the ReliefF algorithm reveals that the overall performance of the conventional liquid cooling system is more influenced by changes in the applied frequency, whereas the novel liquid cooling system’s performance is more sensitive to variations in the water flow rate. These results suggest that sensitivity analysis is more relevant (the dominance of water flow rate) when targeting optimal performance at a specific operating point [52], while ReliefF analysis is more applicable (the dominance of applied frequency over the conventional liquid cooling system and the water flow rate over the novel liquid cooling systems) when designing an optimized system intended to operate efficiently across a wide range of conditions [34,35]. This ML-driven approach significantly reduces the computational burden, eliminating the need for a dedicated FEM simulation for every design point and enabling faster design cycles.

It is important to acknowledge the prominent limitations of the study. The simulations assumed a steady and uniform flow of the cooling liquid, which may not fully represent the complexities of real-world flow dynamics, such as turbulence or variations in flow rate [53]. Also, keeping the total channel surface area fixed when varying the tube radius is theoretically crucial for better thermal comparisons, but this approach was not preferred in our study. The coil wire length, and thus the available channel length, was fixed to preserve the electromagnetic characteristics of the coil, such as magnetic field distribution and intensity. Adjusting channel length to equalize surface area would disrupt these properties and compromise the system’s therapeutic effectiveness. Therefore, thermal optimization was carried out within the constraints of a fixed electromagnetic design. Furthermore, while the ML models demonstrated high predictive accuracy within the studied parameter range, their predictive capability is inherently limited by the scope of the training data [31,33]. This study’s focus on a specific coil geometry and operating conditions also limits the generalizability of the findings to other coil designs or operational scenarios. To address these limitations, future work should primarily focus on experimental validation, representing an important next step in the ongoing development and refinement of this approach.

## 5. Conclusions

This study successfully developed and evaluated three novel liquid cooling systems for RF coils in MH using FEM simulations and ML. Electromagnetic benchmarking confirmed that microchannels with radii of 0.25, 0.30, and 0.35 mm maintained the electromagnetic behavior of solid wires, thereby validating the strategic application of the skin effect in this novel cooling system design. Thermal benchmarking demonstrated a significant performance improvement over conventional liquid cooling systems, with the novel liquid cooling systems achieving lower spatial maximum temperatures, demonstrating the potential for significantly enhanced thermal management in MH RF coils. The performance index predicted via the GPR algorithm further corroborated the superiority of the novel liquid cooling systems, particularly with 0.35 mm microchannels, highlighting an optimal balance between heat removal and energy efficiency. Leveraging uncertainty quantification, the GPR algorithm accurately predicted system behavior, enabling pre-manufacturing design optimization and precise forecasting of performance deviations under experimental conditions. To the best of our knowledge, both the implementation of the microchannel-structured liquid cooling system and the application of ML for cooling system optimization represent novel contributions to the field of MH RF coil cooling system design, offering the potential for substantial advancements in performance and efficiency. The proposed novel liquid cooling system offers a promising pathway to mitigate RF coil heating, potentially enhancing the efficacy and safety of MH treatment. In conclusion, the promising performance of the novel liquid cooling system suggests its adaptability in other RF applications operating at higher frequencies, broadening the significance of this study for future advancements in thermal management across a range of RF technologies.

## Figures and Tables

**Table 1:** Key simulation results for the spatial maximum temperature, smT (°C), in coil wires presented for all four liquid cooling systems.

Applied Frequency (MHz)	Cooling Systems	Water Flow Rate (m/s)	
		0.1	0.5
0.1	Conventional Liquid	**24.964**	**23.172**
	Novel Liquid with 0.25 mm Microchannel Radius	21.590	20.773
	Novel Liquid with 0.30 mm Microchannel Radius	21.376	20.667
	Novel Liquid with 0.35 mm Microchannel Radius	21.275	20.618
2.9	Conventional Liquid	**52.050**	**40.816**
	Novel Liquid with 0.25 mm Microchannel Radius	30.948	25.374
	Novel Liquid with 0.30 mm Microchannel Radius	29.319	24.550
	Novel Liquid with 0.35 mm Microchannel Radius	28.134	23.960

**Table 2:** The performance index, PI (unitless), for the conventional liquid cooling system.

Applied Frequency (MHz)	Water Flow Rate (m/s)				
	0.1	0.2	0.3	0.4	0.5
0.1	**0.879**	**0.736**	0.627	0.544	**0.480**
0.3	**0.839**	**0.727**	0.631	0.555	0.493
0.5	**0.831**	0.736	0.646	0.572	0.511
0.7	0.834	0.750	0.664	0.591	0.531
0.9	0.843	0.767	0.684	0.611	0.551
1.1	0.855	0.785	0.704	0.632	0.572
1.3	0.868	0.805	0.725	0.654	0.592
1.5	0.883	0.824	0.747	0.675	0.613
1.7	0.899	0.845	0.768	0.697	0.634
1.9	0.915	0.865	0.790	0.718	0.655
2.1	0.932	0.886	0.811	0.739	0.675
2.3	0.949	0.906	0.833	0.761	0.696
2.5	0.966	0.927	0.854	0.782	0.716
2.7	0.983	0.947	0.875	0.803	0.737
2.9	**1.000**	0.968	0.896	0.824	0.757

**Table 3:** The performance index, PI (unitless), for the novel liquid cooling system with a 0.25 mm microchannel radius.

Applied Frequency (MHz)	Water Flow Rate (m/s)				
	0.1	0.2	0.3	0.4	0.5
0.1	0.970	0.789	0.662	0.569	**0.499**
0.3	0.992	0.822	0.695	0.601	0.528
0.5	1.026	0.860	0.731	0.634	0.559
0.7	1.063	0.900	0.769	0.668	0.590
0.9	1.103	0.940	0.806	0.702	0.621
1.1	1.143	0.981	0.844	0.737	0.652
1.3	1.183	1.022	0.882	0.771	0.683
1.5	1.224	1.063	0.919	0.805	0.714
1.7	1.264	1.103	0.957	0.839	0.745
1.9	1.305	1.144	0.994	0.873	0.776
2.1	1.345	1.184	1.032	0.907	0.807
2.3	1.386	1.224	1.069	0.941	0.838
2.5	1.426	1.264	1.106	0.975	0.868
2.7	1.466	1.304	1.143	1.008	0.899
2.9	**1.505**	1.344	1.179	1.042	0.930

**Table 4:** The performance index, PI (unitless), for the novel liquid cooling system with a 0.30 mm microchannel radius.

Applied Frequency (MHz)	Water Flow Rate (m/s)				
	0.1	0.2	0.3	0.4	0.5
0.1	0.976	0.792	0.664	0.570	**0.500**
0.3	1.007	0.829	0.699	0.603	0.530
0.5	1.044	0.870	0.737	0.638	0.561
0.7	1.086	0.912	0.776	0.673	0.593
0.9	1.129	0.955	0.815	0.708	0.625
1.1	1.173	0.998	0.854	0.743	0.657
1.3	1.217	1.041	0.893	0.779	0.689
1.5	1.261	1.084	0.932	0.814	0.720
1.7	1.306	1.126	0.971	0.849	0.752
1.9	1.350	1.169	1.010	0.884	0.784
2.1	1.393	1.212	1.049	0.919	0.815
2.3	1.437	1.254	1.088	0.954	0.847
2.5	1.480	1.296	1.126	0.989	0.878
2.7	1.523	1.338	1.165	1.023	0.910
2.9	**1.566**	1.380	1.203	1.058	0.941

**Table 5:** The performance index, PI (unitless), for the novel liquid cooling system with a 0.35 mm microchannel radius.

Applied Frequency (MHz)	Water Flow Rate (m/s)				
	0.1	0.2	0.3	0.4	0.5
0.1	0.980	0.794	0.664	0.571	**0.500**
0.3	1.019	0.835	0.703	0.606	0.532
0.5	1.059	0.877	0.742	0.641	0.563
0.7	1.103	0.921	0.781	0.677	0.596
0.9	1.150	0.965	0.822	0.712	0.628
1.1	1.196	1.010	0.862	0.749	0.660
1.3	1.243	1.055	0.902	0.784	0.693
1.5	1.290	1.099	0.942	0.820	0.725
1.7	1.337	1.144	0.982	0.856	0.757
1.9	1.384	1.188	1.022	0.892	0.790
2.1	1.430	1.232	1.062	0.928	0.822
2.3	1.477	1.276	1.102	0.963	0.854
2.5	1.523	1.320	1.141	0.999	0.886
2.7	1.569	1.364	1.181	1.034	0.918
2.9	**1.614**	1.407	1.220	1.070	0.950

**Table 6:** Adjusted R^2^ error metric calculations for SVR- and GPR-based model predictions of the applied frequency, $f_{a}$, and water flow rate, $v_{wf}$, vs. the performance index, PI, across all liquid cooling systems.

Cooling Systems	SVR	GPR (95% Confidence Level)
Conventional Liquid	0.9777	0.9999
Novel Liquid with 0.25 mm Microchannel Radius	0.9705	0.9999
Novel Liquid with 0.30 mm Microchannel Radius	0.9695	0.9999
Novel Liquid with 0.35 mm Microchannel Radius	0.9687	0.9999

**Table 7:** Sensitivity analysis of input parameter influence on GPR model predictions, and ReliefF feature weights indicating input parameter importance for predictions.

Cooling Systems	Parameter	GPR	
		Sensitivity Analysis	RelifF Feature Weights
Conventional Liquid	Applied Frequency	0.0161	2
	Water Flow Rate	0.0197	1
Novel Liquid with 0.25 mm Microchannel Radius	Applied Frequency	0.0291	1
	Water Flow Rate	0.0319	2
Novel Liquid with 0.30 mm Microchannel Radius	Applied Frequency	0.0308	1
	Water Flow Rate	0.0335	2
Novel Liquid with 0.35 mm Microchannel Radius	Applied Frequency	0.0322	1
	Water Flow Rate	0.0348	2

## Data Availability

The data presented in this study and Supplementary Materials are available on request from the corresponding author.

## Supplementary Materials

The following supporting information can be downloaded at: https://www.mdpi.com/article/10.3390/bioengineering12050490/s1, Table S1: Material properties; Table S2: Spatial maximum current density normal data; Figure S1: Spatial maximum current density normal map at 0.1 MHz; Figure S2: Spatial maximum current density normal map at 1.5 MHz; Figure S3: Spatial maximum current density normal map at 2.9 MHz; Figure S4: Data and SVR model predictions normalized spatial maximum current density normal; Figure S5: Data and GPR model predictions normalized spatial maximum current density normal; Table S3: Error metrics for model predictions of normalized spatial maximum current density normal; Figure S6: Power consumption across the frequency range; Table S4: Spatial maximum temperature data for conventional liquid cooling system; Table S5: Spatial maximum temperature data for novel liquid cooling system with 0.25 mm microchannel radius; Table S6: Spatial maximum temperature data for novel liquid cooling system with 0.30 mm microchannel radius; Table S7: Spatial maximum temperature data for novel liquid cooling system with 0.35 mm microchannel radius; Figure S7: Spatial maximum temperature map at 0.1 MHz and 0.1 m/s; Figure S8: Spatial maximum temperature map at 2.9 MHz and 0.1 m/s; Figure S9: Spatial maximum temperature map at 0.1 MHz and 0.5 m/s; Figure S10: Spatial maximum temperature map at 2.9 MHz and 0.5 m/s; Figure S11: Data and SVR model predictions normalized spatial maximum temperature; Figure S12: Data and GPR model predictions normalized spatial maximum temperature; Table S8: Error metrics for model predictions of normalized spatial maximum temperature; Figure S13: Data and SVR model predictions performance index; Table S9: Sensitivity analysis and ReliefF feature weights for SVR model predictions performance index.
