# Supplementary material for: Simulation-Based Design and Machine Learning Optimization of a Novel Liquid Cooling System for Radio Frequency Coils in Magnetic Hyperthermia
Source: Bioengineering (Basel). 2025 May 4;12(5):490. doi: 10.3390/bioengineering12050490 (PMC12109527; doi:10.3390/bioengineering12050490)
Supplement: Supplementary file 1 [file bioengineering-12-00490-s001.zip › bioengineering-3600883-supplementary.pdf]

Supplementary Materials

Table S1 Simulation material properties for all cooling systems

**Material**	**Property**	**Cooling Systems**
Copper	Relative Permeability	999.994×10^{-3}
	Relative Permittivity	1
	Electrical Conductivity (S/m)	5.87×10^{7}
	Heat Capacity at Constant Pressure ($J.kg^{-1}.K^{-1}$)	385
	Density (kg/m)	8960
	Thermal Conductivity ($W.m^{-1}.K^{-1}$)	401
Air	Relative Permeability	1
	Relative Permittivity	1
	Electrical Conductivity (S/m)	0
	Heat Capacity at Constant Pressure ($J.kg^{-1}.K^{-1}$)	1005
	Specific Gas Constant ($J.kg^{-1}.K^{-1}$)	287
	Thermal Conductivity ($W.m^{-1}.K^{-1}$)	1
Water	Relative Permeability	0.99
	Relative Permittivity	80.1
	Electrical Conductivity (S/m)	0
	Heat Capacity at Constant Pressure ($J.kg^{-1}.K^{-1}$)	4184
	Density (kg/m)	997
	Thermal Conductivity ($W.m^{-1}.K^{-1}$)	0.598
	Ratio of Specific Heats	1

All values were sourced from Total Materia database

A. Electromagnetic Benchmarking

Table S2 Simulation spatial maximum current density normal, *smJ* ($\times 10^8$ A/m^2) in coil wires for all cooling systems

Applied Frequency (MHz)	Cooling Systems				
	Conventional Passive	Conventional Liquid	Novel Liquid with 0.25 mm Microchannel Radius	Novel Liquid with 0.30 mm Microchannel Radius	Novel Liquid with 0.35 mm Microchannel Radius
0.1	1.430	1.433	1.375	1.351	1.347
0.3	2.524	2.531	2.532	2.525	2.482
0.5	3.283	3.293	3.284	3.291	3.295
0.7	3.901	3.913	3.900	3.902	3.912
0.9	4.435	4.449	4.435	4.435	4.439
1.1	4.914	4.929	4.914	4.913	4.914
1.3	5.350	5.366	5.350	5.350	5.349
1.5	5.754	5.772	5.754	5.754	5.753
1.7	6.132	6.151	6.132	6.132	6.131
1.9	6.488	6.508	6.488	6.488	6.488
2.1	6.826	6.847	6.826	6.826	6.826
2.3	7.149	7.171	7.149	7.149	7.149
2.5	7.485	7.489	7.485	7.485	7.485
2.7	7.816	7.820	7.816	7.816	7.816
2.9	8.135	8.140	8.135	8.135	8.135

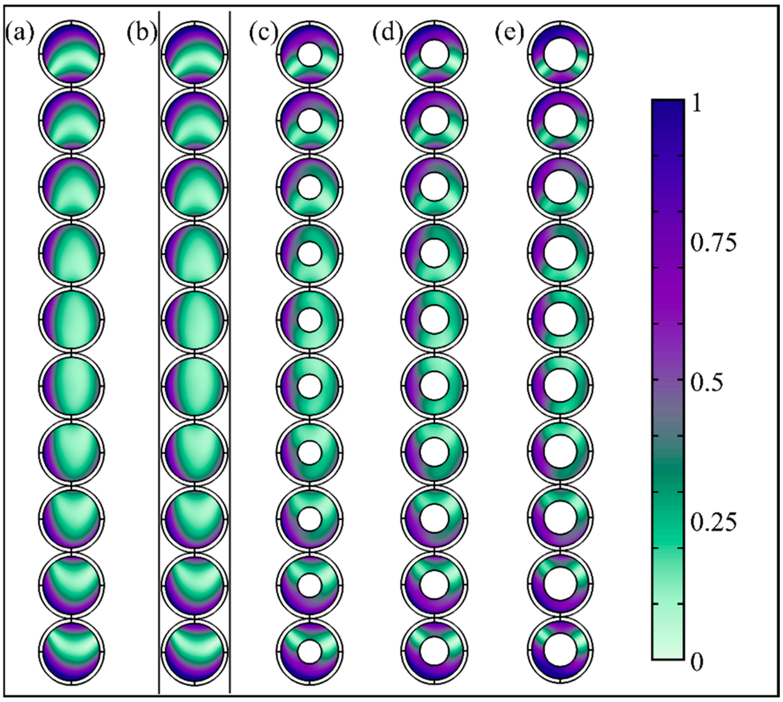

Fig. S1 Simulation results showing 2D slice plots of the spatial maximum current density normal, *smJ* in coil wires at 0.1 MHz applied frequency for all cooling systems; (a) conventional passive, (b) conventional liquid, and (c–e) novel liquid with microchannel radii of 0.25, 0.30, and 0.35 mm, respectively. (The color bar on the right indicates the magnitude of current density normal $\times 10^8$ A/m^2)

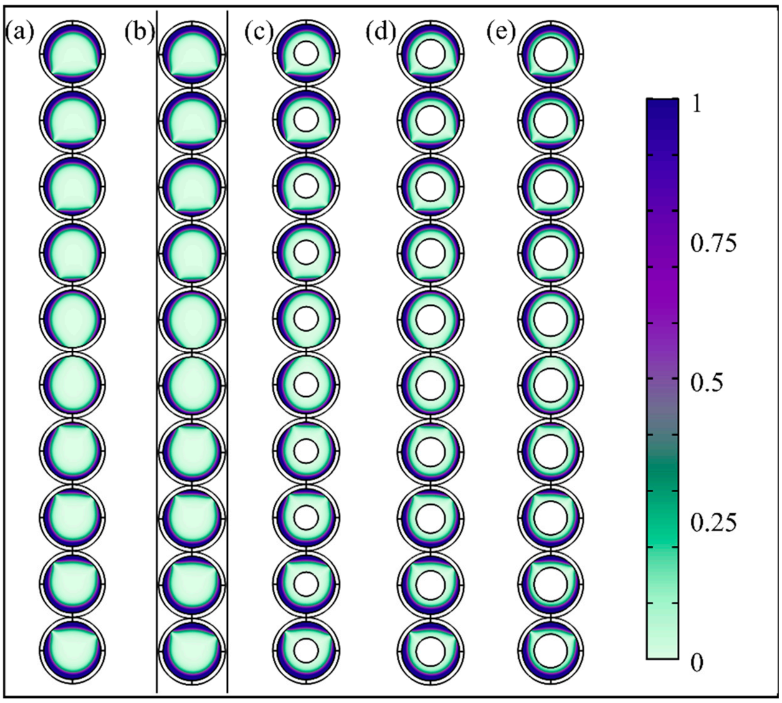

Fig. S2 Simulation results showing 2D slice plots of the spatial maximum current density normal, *smJ* in coil wires at 1.5 MHz applied frequency for all cooling systems; (a) conventional passive, (b) conventional liquid, and (c–e) novel liquid with microchannel radii of 0.25, 0.30, and 0.35 mm, respectively. (The color bar on the right indicates the magnitude of current density normal $\times 10^8$ A/m^2)

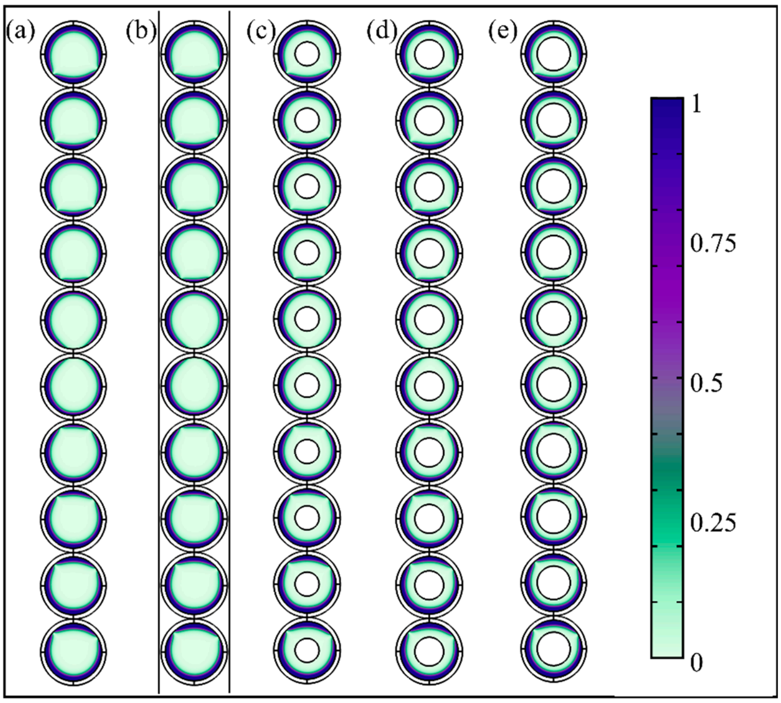

Fig. S3 Simulation results showing 2D slice plots of the spatial maximum current density normal, *smJ* in coil wires at 2.9 MHz applied frequency for all cooling systems; (a) conventional passive, (b) conventional liquid, and (c–e) novel liquid with microchannel radii of 0.25, 0.30, and 0.35 mm, respectively. (The color bar on the right indicates the magnitude of current density normal $\times 10^8$ A/m^2)

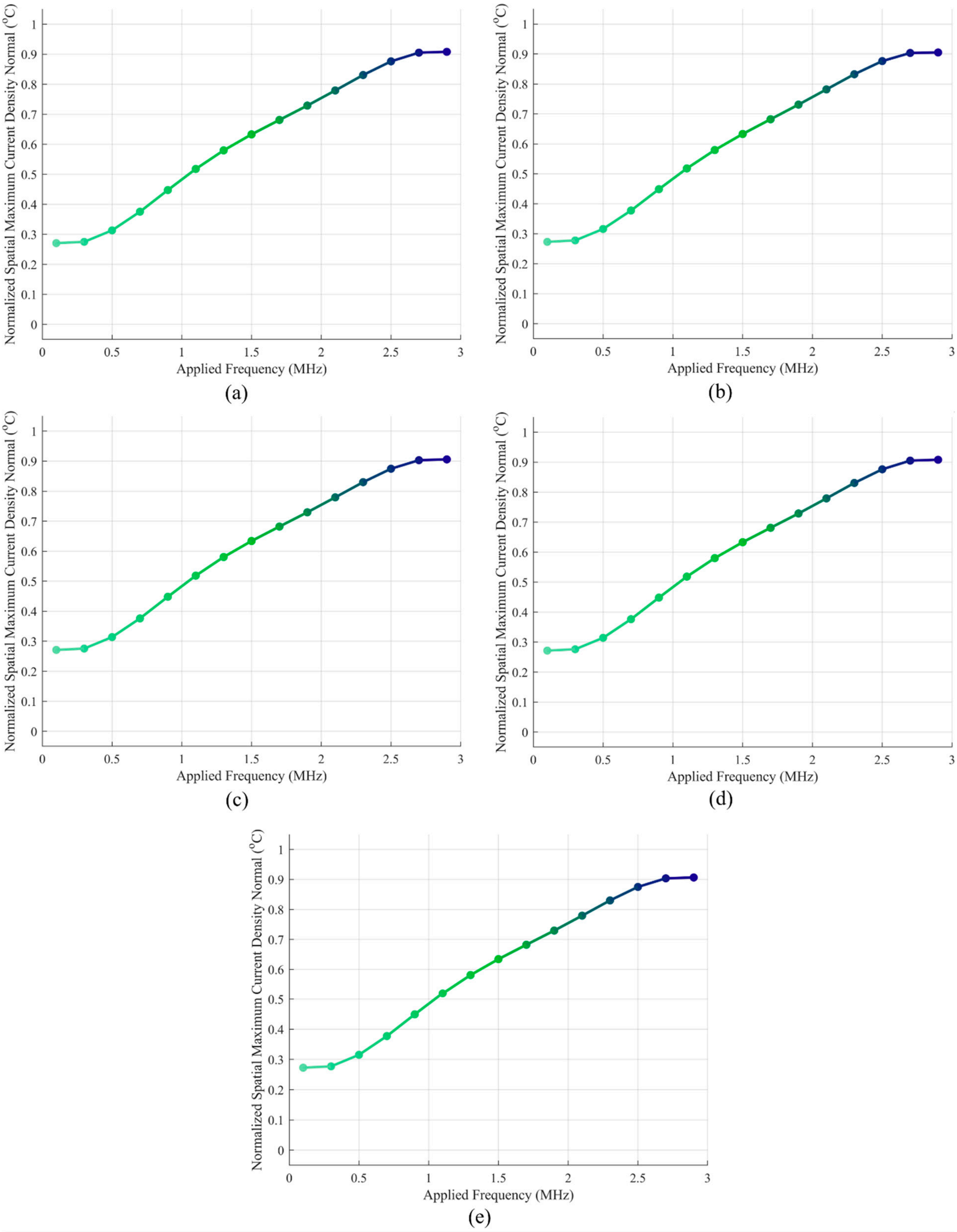

Fig. S4 Data and SVR model predictions of applied frequency, f_a vs. normalized spatial maximum current density normal, $nsmJ$ for various cooling systems: (a) conventional passive, (b) conventional liquid, and (c–e) novel liquid with microchannel radii of 0.25, 0.30, and 0.35 mm, respectively (Scatter: data, line: predicted)

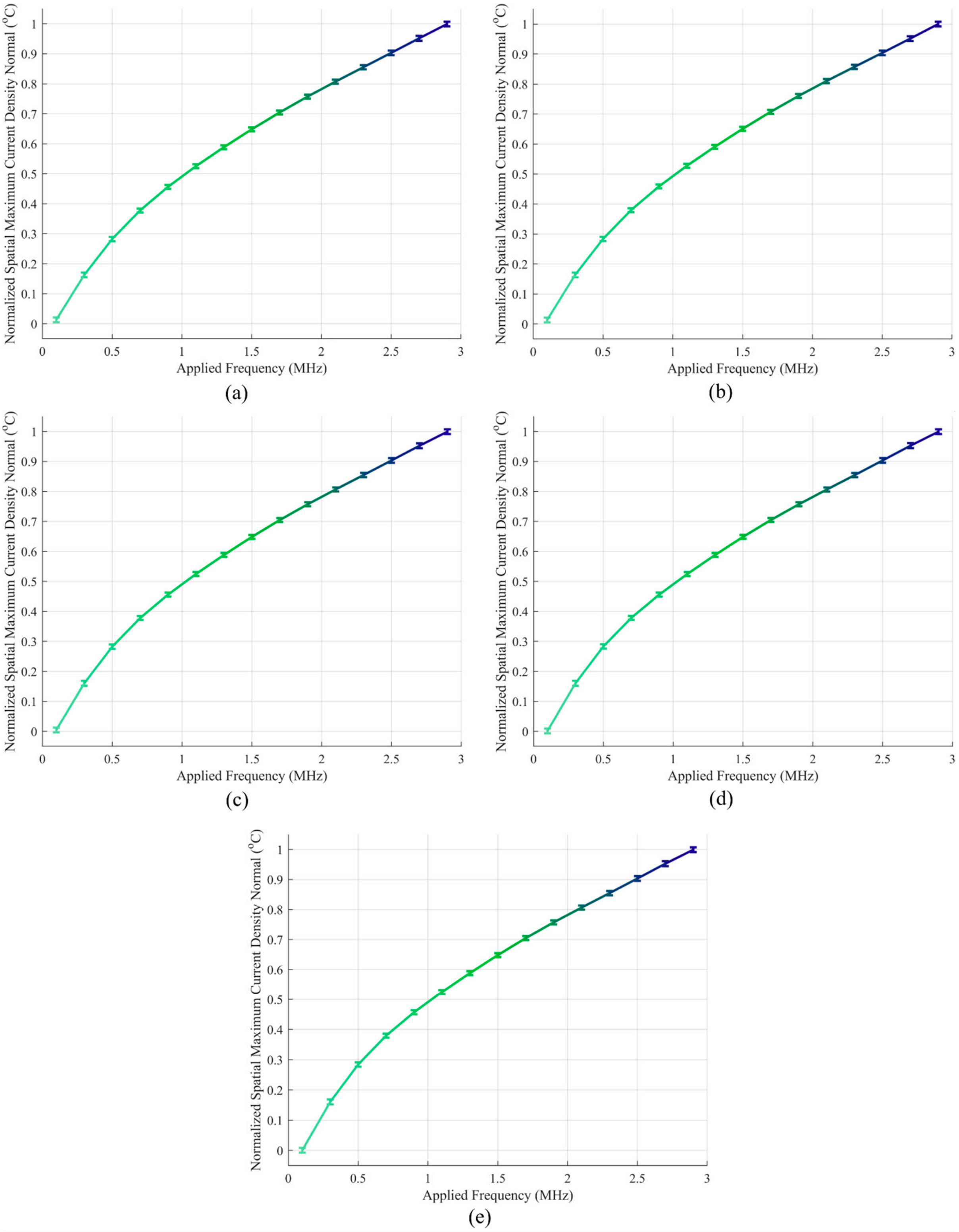

Fig. S5 Data and GPR model predictions of applied frequency, f_a vs. normalized spatial maximum current density normal, $nsmJ$ for various cooling systems: (a) conventional passive, (b) conventional liquid, and (c–e) novel liquid with microchannel radii of 0.25, 0.30, and 0.35 mm, respectively. (Error bars: uncertainty estimates of the predicted data at a 95% confidence level, line: predicted)

Table S3 Adjusted R^2 error metric calculations for model predictions of applied frequency, f_a vs. normalized spatial maximum current density normal, $nsmJ$ across all cooling systems

Cooling Systems	SVR	GPR (95% Confidence Level)
Conventional Passive	0.9272	0.9994
Conventional Liquid	0.9235	0.9994
Novel Liquid with 0.25 mm Microchannel Radius	0.9263	0.9989
Novel Liquid with 0.30 mm Microchannel Radius	0.9261	0.9990
Novel Liquid with 0.35 mm Microchannel Radius	0.9177	0.9998

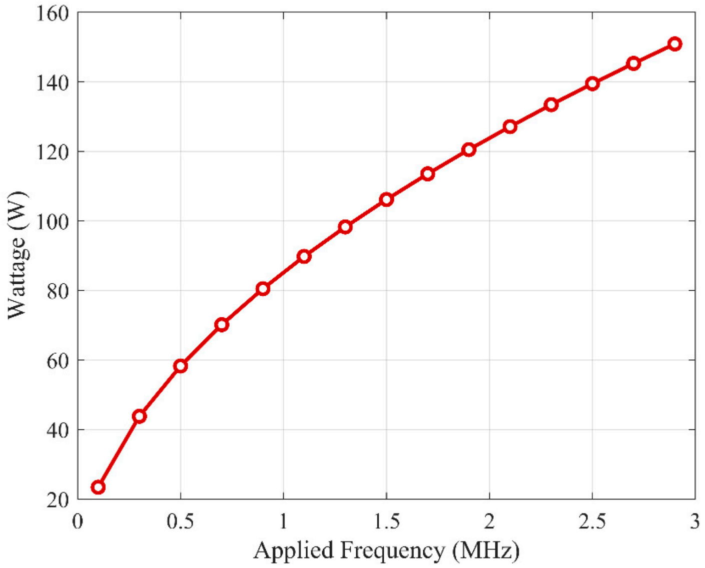

Fig. S6 Computed coil power consumption across the range of applied frequencies for all design configurations. (Single line is used to represent all designs, as their wattage values were nearly identical across the frequency range)

B. Thermal Benchmarking

Table S4 Simulation spatial maximum temperature, smT (°C) in coil wires for the conventional liquid cooling system

Applied Frequency (MHz)	Water Flow Rate (m/s)				
	0.1	0.2	0.3	0.4	0.5
0.1	24.964	24.050	23.621	23.359	23.172
0.3	29.317	27.621	26.827	26.340	26.007
0.5	32.398	30.153	29.092	28.454	28.016
0.7	34.928	32.231	30.967	30.191	29.663
0.9	37.131	34.037	32.592	31.700	31.096
1.1	39.102	35.657	34.046	33.058	32.384
1.3	40.906	37.140	35.382	34.293	33.556
1.5	42.577	38.514	36.609	35.442	34.644
1.7	44.141	39.797	37.765	36.516	35.664
1.9	45.616	41.009	38.854	37.531	36.628
2.1	47.018	42.158	39.888	38.493	37.541
2.3	48.355	43.257	40.873	39.410	38.414
2.5	49.634	44.307	41.818	40.287	39.243
2.7	50.865	45.312	42.727	41.128	40.047
2.9	52.050	46.286	43.600	41.946	40.816

Table S5 Simulation spatial maximum temperature data, smT (°C) in coil wires for the novel liquid cooling system with 0.25 mm cooling microchannel radius

Applied Frequency (MHz)	Water Flow Rate (m/s)				
	0.1	0.2	0.3	0.4	0.5
0.1	21.590	21.171	20.976	20.856	20.773
0.3	23.134	22.311	21.927	21.692	21.528
0.5	24.210	23.106	22.591	22.274	22.054
0.7	25.081	23.751	23.129	22.747	22.482
0.9	25.840	24.313	23.598	23.160	22.854
1.1	26.521	24.817	24.019	23.529	23.189
1.3	27.141	25.277	24.404	23.867	23.494
1.5	27.715	25.702	24.759	24.180	23.777
1.7	28.251	26.100	25.092	24.472	24.041
1.9	28.756	26.475	25.405	24.748	24.290
2.1	29.235	26.831	25.703	25.009	24.527
2.3	29.691	27.169	25.986	25.259	24.752
2.5	30.127	27.493	26.257	25.497	24.968
2.7	30.545	27.804	26.517	25.726	25.175
2.9	30.948	28.104	26.768	25.946	25.374

Table S6 Simulation spatial maximum temperature, smT (°C) in coil wires for the novel liquid cooling system with 0.30 mm cooling microchannel radius

Applied Frequency (MHz)	Water Flow Rate (m/s)				
	**0.1**	**0.2**	**0.3**	**0.4**	**0.5**
0.1	21.376	21.012	20.842	20.739	20.667
0.3	22.636	21.940	21.616	21.417	21.280
0.5	23.582	22.637	22.197	21.927	21.740
0.7	24.324	23.184	22.653	22.328	22.102
0.9	24.967	23.659	23.049	22.675	22.416
1.1	25.544	24.085	23.405	22.988	22.698
1.3	26.073	24.476	23.730	23.274	22.957
1.5	26.562	24.837	24.032	23.539	23.196
1.7	27.019	25.175	24.314	23.787	23.420
1.9	27.450	25.494	24.580	24.020	23.631
2.1	27.858	25.796	24.832	24.242	23.832
2.3	28.247	26.083	25.073	24.453	24.023
2.5	28.619	26.359	25.303	24.655	24.205
2.7	28.976	26.623	25.524	24.850	24.381
2.9	29.319	26.878	25.737	25.037	24.550

Table S7 Simulation spatial maximum temperature, smT (°C) in coil wires for the novel liquid cooling system with 0.35 mm cooling microchannel radius

Applied Frequency (MHz)	Water Flow Rate (m/s)				
	**0.1**	**0.2**	**0.3**	**0.4**	**0.5**
0.1	21.275	20.936	20.780	20.684	20.618
0.3	22.245	21.650	21.374	21.206	21.089
0.5	23.104	22.282	21.901	21.668	21.506
0.7	23.776	22.777	22.313	22.029	21.833
0.9	24.339	23.192	22.658	22.333	22.106
1.1	24.840	23.560	22.966	22.602	22.350
1.3	25.298	23.898	23.248	22.850	22.574
1.5	25.724	24.213	23.510	23.080	22.782
1.7	26.123	24.507	23.755	23.295	22.976
1.9	26.499	24.785	23.987	23.499	23.160
2.1	26.856	25.048	24.207	23.692	23.335
2.3	27.196	25.299	24.416	23.876	23.501
2.5	27.521	25.539	24.617	24.052	23.660
2.7	27.833	25.770	24.809	24.221	23.813
2.9	28.134	25.992	24.994	24.384	23.960

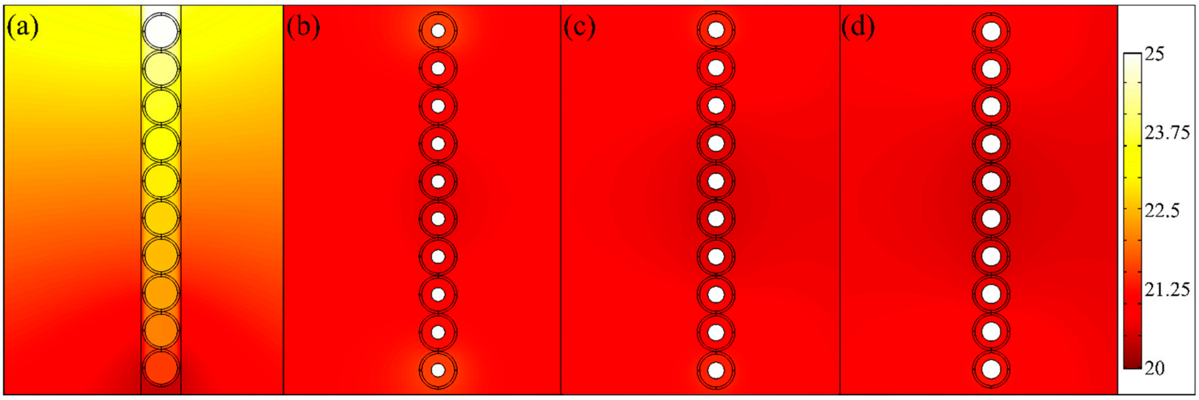

Fig. S7 Simulation results showing 2D slice plots of the spatial maximum temperature, *smT* in and around coil wires at 0.1 MHz applied frequency and 0.1 m/s water flow rate for liquid cooling systems; (a) conventional liquid, and (b–d) novel liquid with microchannel radii of 0.25, 0.30, and 0.35 mm, respectively. (The color bar on the right indicates the magnitude of temperature °C)

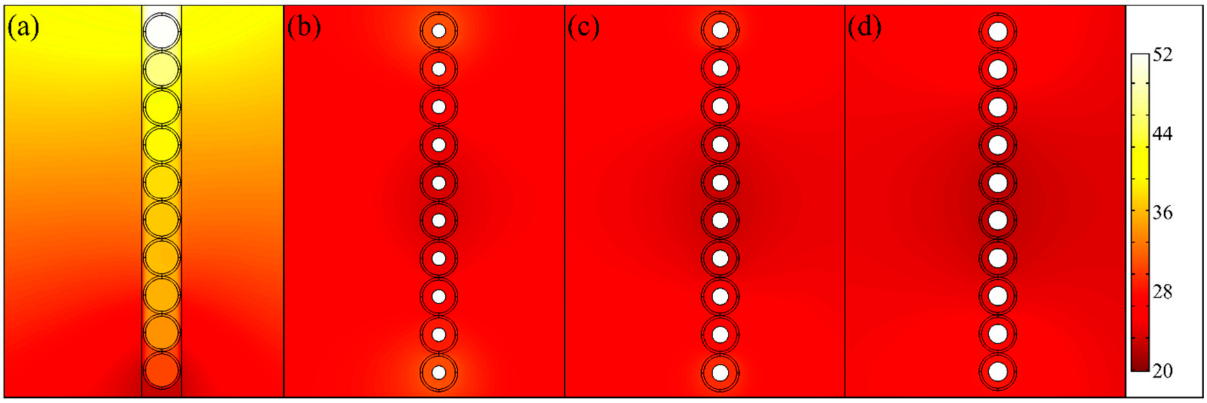

Fig. S8 Simulation results showing 2D slice plots of the spatial maximum temperature, *smT* in and around coil wires at 2.9 MHz applied frequency and 0.1 m/s water flow rate for liquid cooling systems; (a) conventional liquid, and (b–d) novel liquid with microchannel radii of 0.25, 0.30, and 0.35 mm, respectively. (The color bar on the right indicates the magnitude of temperature °C)

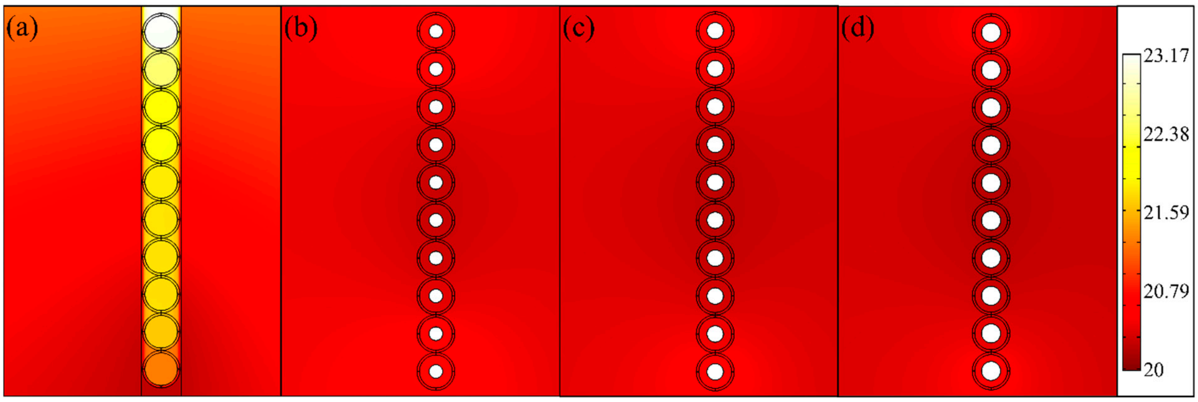

Fig. S9 Simulation results showing 2D slice plots of the spatial maximum temperature, *smT* in and around coil wires at 0.1 MHz applied frequency and 0.5 m/s water flow rate for liquid cooling systems; (a) conventional liquid, and (b–d) novel liquid with microchannel radii of 0.25, 0.30, and 0.35 mm, respectively. (The color bar on the right indicates the magnitude of temperature °C)

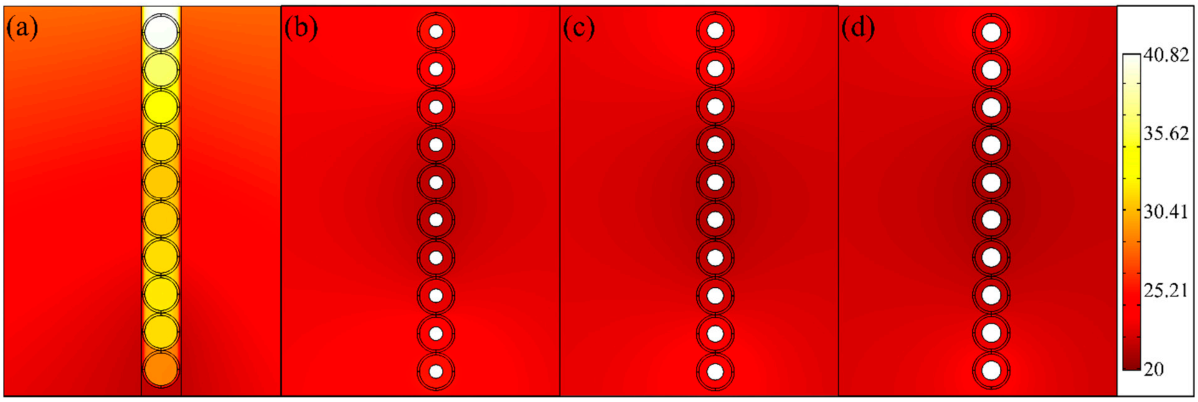

Fig. S10 Simulation results showing 2D slice plots of the spatial maximum temperature, smT in and around coil wires at 2.9 MHz applied frequency and 0.5 m/s water flow rate for liquid cooling systems; (a) conventional liquid, and (b–d) novel liquid with microchannel radii of 0.25, 0.30, and 0.35 mm, respectively. (The color bar on the right indicates the magnitude of temperature °C)

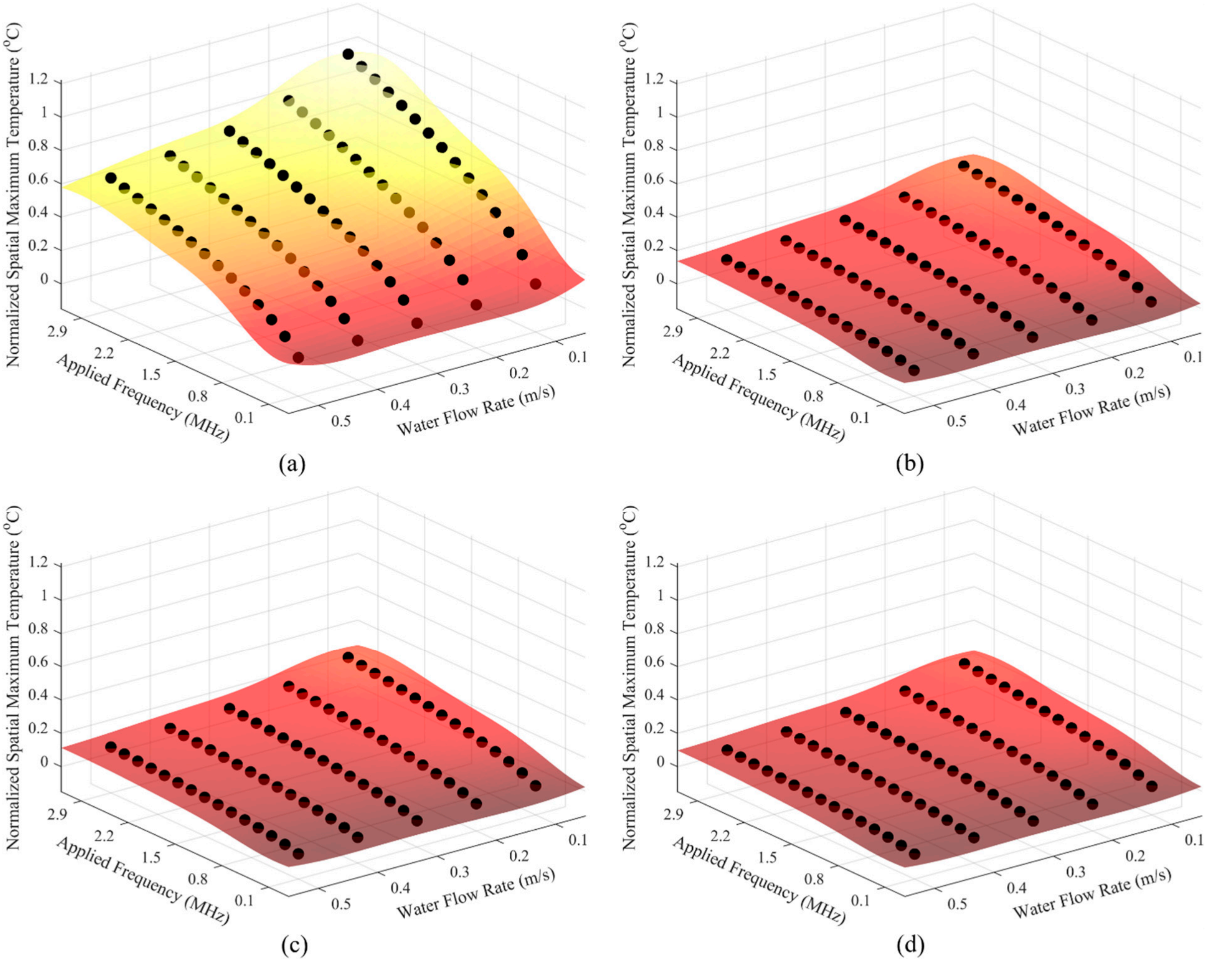

Fig. S11 Data and SVR model predictions of applied frequency, f_a – water flow rate, v_{wf} vs. normalized spatial maximum temperature, $nsmT$ for all liquid cooling systems: (a) conventional liquid, and (c–d) novel liquid with microchannel radii of 0.25, 0.30, and 0.35 mm, respectively. (Scatter: data, surface: predicted)

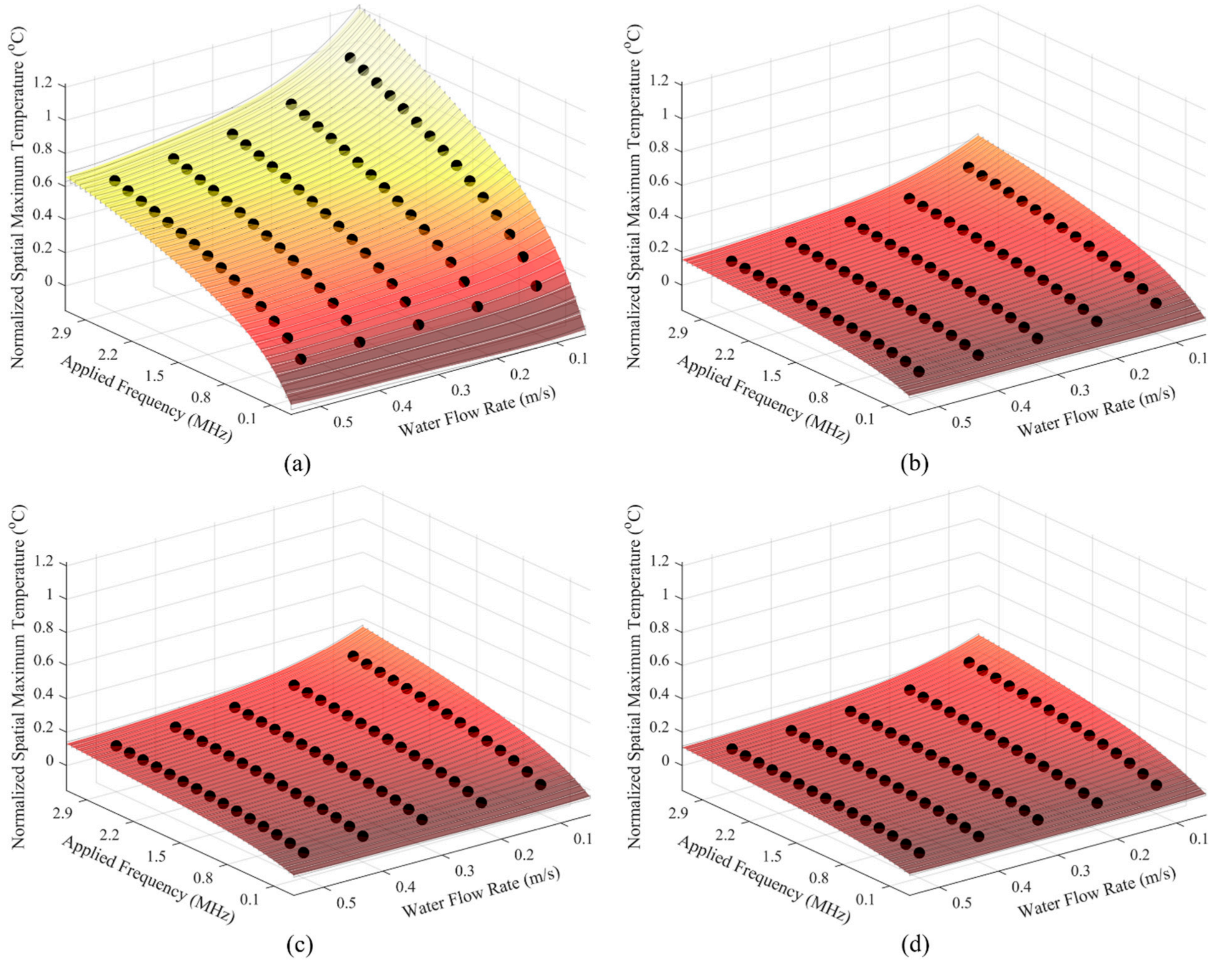

Fig. S12 Data and GPR model predictions of applied frequency, f_a – water flow rate, v_{wf} vs. normalized spatial maximum temperature, $nsmT$ for all liquid cooling systems: (a) conventional liquid, and (c–d) novel liquid with microchannel radii of 0.25, 0.30, and 0.35 mm, respectively (Scatter: data, surface: predicted, the lines encircling the color surface indicate uncertainty estimates at a 95% confidence level)

Table S8 Adjusted R^2 error metric calculations for model predictions of applied frequency, f_a – water flow rate, v_{wf} vs. normalized spatial maximum temperature, $nsmT$ across all liquid cooling systems

**Cooling Systems**	**SVR**	**GPR (95% Confidence Level)**
Conventional Liquid	0.9581	0.9999
Novel Liquid with 0.25 mm Microchannel Radius	0.9597	0.9998
Novel Liquid with 0.30 mm Microchannel Radius	0.9593	0.9998
Novel Liquid with 0.35 mm Microchannel Radius	0.9587	0.9999

C. Performance Index

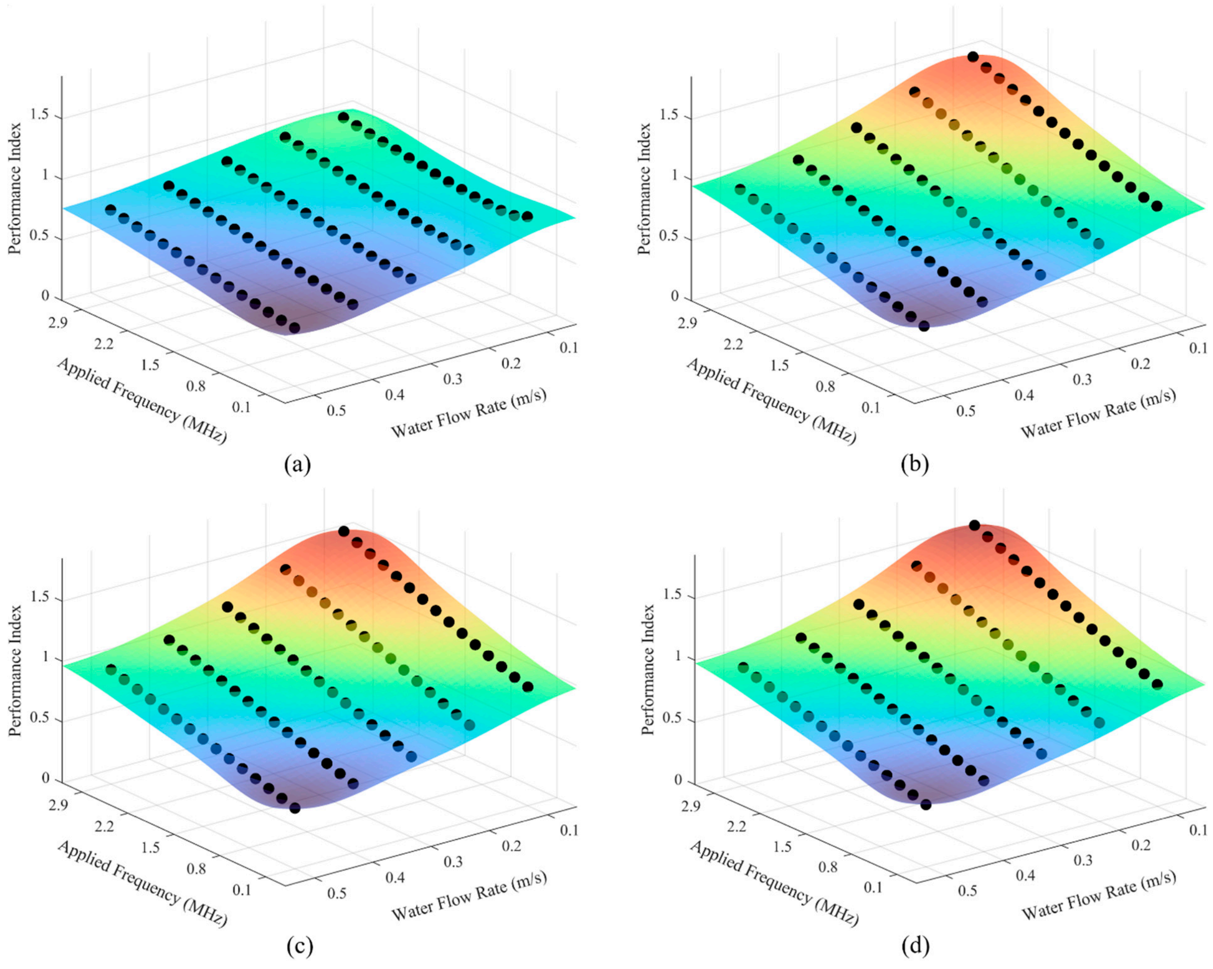

Fig. S13 Data and SVR model predictions of applied frequency, f_a – water flow rate, v_{wf} vs. performance index, PI for all liquid cooling systems: (a) conventional liquid, and (c–d) novel liquid with microchannel radii of 0.25, 0.30, and 0.35 mm, respectively. (Scatter: data, surface: predicted)

Table S9 Sensitivity analysis of input parameter influence on SVR model predictions, and ReliefF feature weights indicating input parameter importance for predictions

**Cooling Systems**	**Parameter**	**SVR**	
		**Sensitivity Analysis**	**ReliffF Feature Weights**
Conventional Liquid	Applied Frequency	0.0161	2
	Water Flow Rate	0.0173	1
Novel Liquid with 0.25 mm Microchannel Radius	Applied Frequency	0.0335	1
	Water Flow Rate	0.0262	2
Novel Liquid with 0.30 mm Microchannel Radius	Applied Frequency	0.0355	1
	Water Flow Rate	0.0271	2
Novel Liquid with 0.35 mm Microchannel Radius	Applied Frequency	0.0370	1
	Water Flow Rate	0.0277	2
